# Extinct before discovered? *Epactoidesgiganteus* sp. nov. (Coleoptera, Scarabaeidae, Scarabaeinae), the first native dung beetle to Réunion island

**DOI:** 10.3897/zookeys.1061.70130

**Published:** 2021-10-01

**Authors:** Michele Rossini, Fernando Z. Vaz-de-Mello, Olivier Montreuil, Nicholas Porch, Sergei Tarasov

**Affiliations:** 1 Finnish Museum of Natural History (LUOMUS), University of Helsinki, Pohjoinen Rautatiekatu 13, Helsinki, 00014, Finland University of Helsinki Helsinki Finland; 2 Departamento de Biologia e Zoologia, Instituto de Biociências, Universidade Federal de Mato Grosso, Av. Fernando Correa da Costa, n 2367, Boa Esperança, 78060-900, Cuiabá, Mato Grosso, Brazil Universidade Federal de Mato Grosso Cuiabá Brazil; 3 UMR 7179 MNHN/CNRS, MECADEV, Muséum National d’Histoire Naturelle, Entomologie, CP 50, 45 rue Buffon, 75231 Paris cedex 05, France Muséum National d’Histoire Naturelle Paris France; 4 School of Life and Environmental Sciences, Faculty of Science Engineering & Built Environment, Deakin University, Melbourne Burwood Campus, 221 Burwood Highway, Burwood, VIC 3125, Australia Deakin University Melbourne Australia

**Keywords:** Dung beetles, extinction, Madagascar, Malagasy region, Mascarene Archipelago, Nicolas Bréon, over-water dispersal

## Abstract

We describe a new species of dung beetle, *Epactoidesgiganteus***sp. nov.**, from a single female specimen allegedly collected in the 19^th^ century on Réunion island and recently found at the Muséum national d’Histoire naturelle, Paris. This species differs from other species of *Epactoides* by larger size and a set of other distinctive morphological characters. *Epactoidesgiganteus***sp. nov.** is the first native dung beetle (Scarabaeinae) of Réunion, and its discovery expands the known area of distribution of the genus *Epactoides*, which was hitherto believed to be endemic to Madagascar. Like other taxa from Madagascar and peripheral islands (e.g., Comoro, Seychelles, Mascarenes), *E.giganteus***sp. nov.** may have reached Réunion by over-water dispersal. Given the rapid loss of biodiversity on Réunion island and the fact that no additional specimens were re-collected over the last two centuries, it is very likely that *E.giganteus***sp. nov.** has gone extinct. However, we have unconfirmed evidence that the holotype of *E.giganteus***sp. nov.** might be a mislabeled specimen from Madagascar, which would refute the presence of native dung beetles on Réunion. We discuss both hypotheses about the specimen origin and assess the systematic position of *E.giganteus***sp. nov.** by examining most of the described species of Madagascan *Epactoides*. Additionally, we provide a brief overview of the dung beetle fauna of Mascarene Archipelago.

## Introduction

The Mascarene archipelago is located in the southwestern Indian Ocean and comprises three main volcanic isles, namely Réunion, Mauritius, and Rodrigues. At about 2,510 km^2^, Réunion is the largest of the Mascarene islands and the closest to Madagascar (ca 550 km), followed by Mauritius (ca 1865 km^2^) and Rodriguez (ca 110 km^2^), which are situated at an increasing distance from Madagascar, about 900 km and 1,500 km, respectively.

The three Mascarene islands are globally renowned as iconic examples of recent and rapid loss of a great part of their biotas. According to early reports and ecological inferences based on current vegetation, at the time of their discovery, the Mascarene islands were completely covered with dense, high forests (Cheke and Hume 2008). Uncontrolled logging, over-hunting, and accidental and voluntary introduction of alien animals, along with a long list of invasive plants had a devastating effect on local Mascarene biotas. Nowadays, agricultural practices primarily linked to the large production of sugarcane have extensively modified native ecosystems. However, fortunately, the rugged topography of Mauritius and Réunion would seem to have played a crucial role for the survival of small fragments of native and very fragile ecosystems. For example, in Mauritius, many of these areas are today officially protected by conservation actions (e.g., Conservation Management Areas; Griffiths and Florens 2006; Cheke and Hume 2008).

Mauritius was colonized in 1598 and since then 98% of its primary forests and about 40% of native endemic terrestrial fauna disappeared (Florens 2013). Likewise, since the arrival of Europeans in 1665, about 70% of native terrestrial vertebrate fauna of Réunion went extinct, and its flora is nowadays dominated by an extremely high number of invasive plants (Lagabrielle et al. 2011). Rodrigues was the last of the Mascarene islands to have been reached by humans, yet its historical epilogue was not that different from the other two islands. Leguat (1708) provided the first descriptive framework of the natural environments of Rodriguez at the time of its discovery, bearing witness to the drastic environmental changes suffered by the island over the last three centuries (see Strahm 1989).

**Figure 1.**
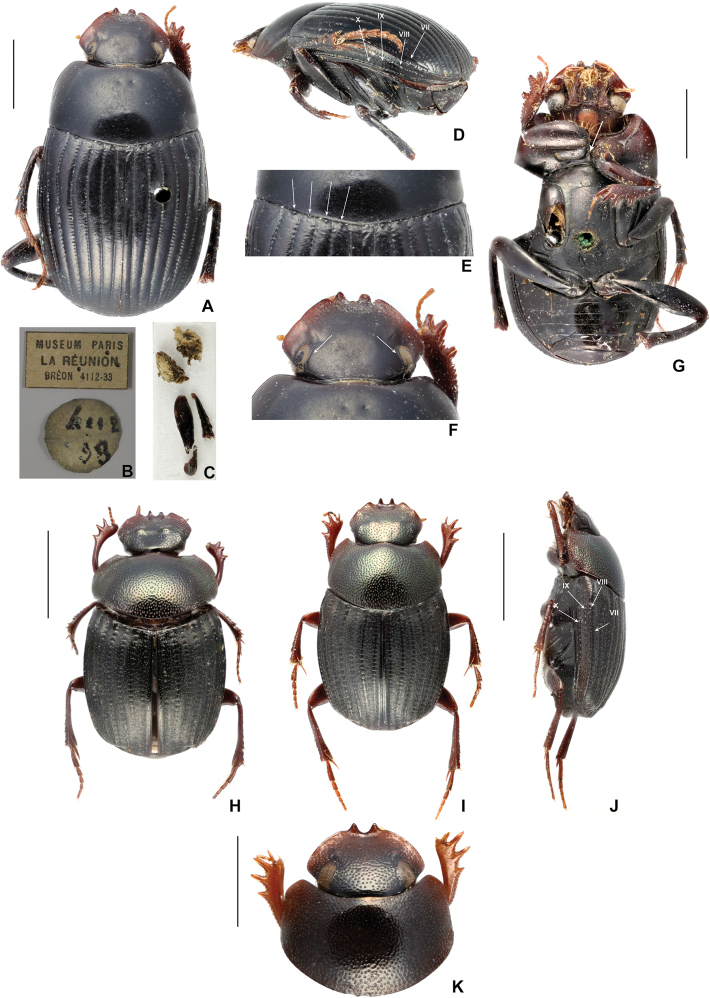
*Epactoidesgiganteus* sp. nov. **A** dorsal habitus of the holotype **B** original labels **C** disarticulated body parts pinned with the holotype **D** detail of the entire carina of the elytral stria 8 and pseudocarina on the apical part of the stria 7; arrows indicate striae 7–10 **E** detail of the base of elytral interstriae; arrows indicate basal tubercles on interstriae 2–5 **F** detail of the dorsal portion of eyes; arrows indicate the internal groove **G** ventral habitus of the holotype; arrow indicates the prosternal spur. *Epactoidesfrontalis* (Montreuil) **H, I** dorsal habitus of male and female **J** lateral habitus of the female; arrows indicate elytral stria 7–10; stria 7 completely carinated. *Ochicanthonceylonicus* Cuccodoro, **K** detail of the wide eyes in dorsal view.

Emblematic examples of lost vertebrate in the Mascarene islands are the dodo (*Rhaphuscucullatus* (Linnaeus, 1758)), the Rodrigues solitaire (*Pezophapssolitaria* (Gmelin, 1789)), day-geckos (genus *Phelsuma* Gray, 1828), giant tortoises (genus *Cylindraspis* Fitzinger, 1835) and fruit bats (genus *Pteropus* Brisson, 1762) (see Griffiths and Florens 2006; Cheke and Hume 2008; Florens 2013). However, very little is known about the invertebrate fauna, and especially insects. As many other oceanic islands (e.g., Antilles, New Caledonia, New Zealand, Madagascar), the Mascarene islands still hold their own endemic taxa and scarabaeine dung beetles are not the exception. Mauritius today harbors two endemic dung beetle genera (i.e., the monotypic *Nesovinsonia* Martínez & Pereira, 1958 and *Nesosisyphus* Vinson, 1946) and a total of five described species; no dung beetles are mentioned from Rodrigues. However, about 12 new subfossil species of scarabaeines have been recently found (Nicholas Porch unpubl. data), which proves that in the past local ecosystems sustained an impressively rich dung beetles fauna. Finally, Réunion seems to host only a few introduced Indian and African *Onthophagus* Latreille, 1802 and some Aphodiinae (Lacroix and Poussereau 2019). Nevertheless, one of us (Fernando Vaz-de-Mello), during a recent visit at the Coleoptera collection of the Muséum national d’Histoire naturelle, Paris, found a gigantic female specimen of *Epactoides* Olsoufieff, 1947 that had two labels: the older label bears the handwritten accession number “4112.33”, which recalls one of the several lots of insects collected and sent by Nicolas Bréon from Réunion; the second, more recent and printed label reads “La Réunion”, including also the collector name and the same accession number (Fig. 1B).

With about 40 species (Fairmaire 1898; Paulian 1935, 1975, 1976, 1991, 1992; Lebis 1953; Montreuil 2003, 2005, 2017; Wirta and Montreuil 2008), the genus *Epactoides* is nowadays considered to be endemic to Madagascar. Most of the described species were formerly included in *Epactoides* and its subgenus Aleiantus (Lebis 1953). Later, new genera were erected to accommodate one or two species each, such as *Sikorantus* Paulian, 1976, *Phacomosoides* Martínez & Pereira, 1958, and *Madaphacosoma* Paulian, 1975. Recently, the phylogenetic analyses by Wirta and Montreuil (2008) has proved the inconsistency of such classification and all these taxon names, including *Aleiantus*, were eventually synonymized under *Epactoides*.

*Epactoides* dung beetles are very small, with body length ranging from 2–5 mm. However, the external phenotype is quite variable: body evenly dark colored or with large and symmetrical yellow spots; dorsal surface of the body polished, shining and punctation very superficial and weak, or body opaque, with deeper and coarse punctation, tubercles, granules, cavities and wrinkles; when present, sexual dimorphisms manifested in the shape of the procoxal cavities (distinctly wider in male), modification of pro- (with medial tooth anteriorly) and metafemora (widened posteriorly), and sometimes in the shape of protibiae (slender and strongly curved apically).

In this study, we describe a new *Epactoides* species from Réunion. The description of the external phenotype is based on a fairly well-preserved female, which is to date the only specimen available to us. The morphological study of a large sample of Madagascan *Epactoides*, the consultation of relevant literature along with historical accession catalogues stored at the Muséum national d’Histoire naturelle, Paris (MNHN), allow us to speculate about the provenance of this gigantic *Epactoides* and to discuss the systematic value and uniqueness of its phenotypic characters.

## Material and methods

The morphological examination of the holotype of the new species was carried out under a Leica S9D stereomicroscope. Photographs of the dorsal habitus and disarticulated body parts were taken with a Canon EOS 5D camera and a Canon MP-E 65 mm, f/2.8, 1–5× macro lens, using the Cognisys Stackshot automated system. Images were subsequently enhanced and edited in Adobe Photoshop and Illustrator CC. Morphological analyses were carried out on specimens deposited in the following institutes:

**MNHN**Muséum national d’Histoire naturelle, Paris (O. Montreuil);

**MZHF** Finnish Zoology Museum of Natural History (LUOMUS), Helsinki (S. Tarasov, J. Mattila).

Original label data are provided *verbatim*; data of different labels are separated by slashes (“/”), while data contained in single label are separated by commas (“,”).

## Results and discussion

### Epactoides giganteus

Taxon classificationAnimaliaColeopteraScarabaeidae

Rossini, Vaz-de-Mello & Montreuil sp. nov.

30EF1052-DDE2-551A-8B6A-12F39F9000A9

http://zoobank.org/CD8ECE33-60B4-4FCA-BE78-CAD7DC56207C

Figure 1A–K

#### Type material.

***Holotype***, female: “MUSEUM PARIS, LA RÉUNION, BRÉON, 4112·33 / 4112, 33 / HOLOTYPE, *Epactoidesgiganteus* Rossini, Vaz-de-Mello, Montreuil, 2021”, (MNHN).

#### Diagnosis.

*Epactoidesgiganteus* sp. nov. is easily distinguished from congeneric species by the uniquely large size (body length 9 mm, while Madagascan *Epactoides* includes only small-sized species, with body length ranging from 2–5 mm); from above, dorsal portion of eyes wide (0.2 mm, while very narrow in Madagascan *Epactoides*); presence of a shallow groove close to the external and internal edges of the eye; elytral stria 7 pseudocarinated, stria 8 entirely carinated (elytral stria 7 entirely carinated in most Madagascan *Epactoides*); presence of a prosternal medial spur (absent in Madagascan *Epactoides*). Also, *E.giganteus* sp. nov. is endemic to and the only known native scarabaeine of Réunion island (but see discussion below). Currently, it is the only species of the genus recorded outside Madagascar.

#### Description.

**Body length.** 9 mm.

**Color.** Dorsal habitus completely black, lateral sides of head and pronotum, and ventral side of body mahogany brown; hairs yellow; mouthparts, tarsi, and antennal articles brownish (antennal club lacking).

***Head*.** Semicircular and barely emarginated in clypeogenal junction; genae finely margined, clypeal edge without margin; clypeus with two median, blunt teeth separated by a wide depression; external side of each clypeal tooth with a deep, V-shaped emargination; dorsal portion of eyes wide (0.2 mm) (Fig. 1A, F); internal and external edges of eyes flanked by a superficial groove that disappears anteriorly; internal groove more distinct and runs from postoccipital margin (Fig. 1F); head largely smooth, with coarse and very shallow punctures in proximity of clypeal teeth.

***Thorax*.** Pronotum feebly convex, lateral and anterior edges finely margined, posterior edge without margin; lateral edges nearly straight and parallel, slightly curved distally; anterior angles rounded; anteromedial region of pronotum with two small, symmetrical pits (Fig. 1A, F); pronotal surface without punctation. Propleuron with a strong and rather thick ridge that runs from lateral margin of procoxae to external margin of pronotum; anterior side of propleuron very slightly excavated. Prosternum smooth, with an anteromedial, blunt spur (Fig. 1G). Meso- and metasternum simple; meso- metasternal junction indicated by a transverse bead, slightly swollen medially. Elytra with 10 striae (two striae on epipleuron); 7^th^ stria pseudocarinated (swollen) from middle to subapical region of elytra; 8^th^ stria completely carinated (Fig. 1D); strial punctures coarse and close; interstriae unpunctated, basomedial margin of interstriae 2–5 with small, rounded tubercles (Fig. 1E).

***Abdomen*.** Sternites well visible ventrally, without punctures and setae; pygidial edges completely margined, basal edge with prominent border; pygidium swollen at middle, highest point of hump connected by two blunt ridges to basal angles of pygidium; abdominal tergite 8^th^ medially interrupted by a deep longitudinal groove.

***Legs*.** Protarsi simple, with 2–3 setae in ventroapical side; mesotarsi long, with a series of aligned setae in ventral and dorsal sides; metatarsi are lacking; protibiae with three big external teeth, externobasal edge serrated; meso- and metatibiae long, straight and distally feebly wider; pro-, meso- and metafemurs unmodified.

#### Distribution.

Réunion island, Mascarene Archipelago (no additional collecting data available).

##### Taxonomically informative characters of *Epactoidesgiganteus* sp. nov.

The external morphology of *E.giganteus* sp. nov. unequivocally indicates its belonging to the genus *Epactoides*: body oval, rather flat dorsoventrally; genae finely margined; clypeus with anteromedial teeth; elytral striae well indicated. This new species is only known from one female specimen, which makes it difficult to suggest any hypothesis of relationships with other *Epactoides*. At the moment, we consider it to be tentatively related to *E.frontalis* (Montreuil, 2003) (Fig. 1H–J) and *E.spinicollis* (Montreuil, 2003). Additional specimens may help to clarify its systematic position.

*Epactoidesgiganteus* sp. nov. exhibits a series of unique phenotypic characters within the genus:

Clypeus with four teeth: this character does not occur in any other *Epactoides* species. Among the Madagascan *Epactoides* examined in this study, *E.frontalis*, *E.spinicollis*, *E.semiaeneus* (Paulian, 1976), and *E.mesoalae* (Paulian, 1976) are the only Madagascan species whose clypeal shape may recall that of *E.giganteus* sp. nov., albeit the lateral clypeal teeth are not as distinctly shaped as in *E.giganteus* sp. nov. (Fig. 1A, F).

Dorsal portion of eyes wide: this phenotypic trait is unique within *Epactoides*, as the eyes of all described species are narrow. Recent phylogenetic reconstructions (Wirta et al. 2010; Mlambo et al. 2014) consider *Epactoides* to be a close relative to the Oriental genus *Ochicanthon* Vaz-de-Mello, 2003, which includes several species with wide eyes (see for example *Ochicanthonceylonicus* Cuccodoro, 2011; Fig. 1K).

Distinct furrow in the inner side of the eyes connected with the postoccipital margin of the head (Fig. 1F): none of the analyzed *Epactoides* species exhibits this character. Similar furrow is common in the Madagascan dung beetle genus *Apotolamprus* Olsoufieff, 1947, where, however, the groove is deeper and never connected with the postoccipital margin of the head.

Elytra with 10 distinct striae; elytral stria 7 pesudocarinated from the middle to the subapical region of the elytra; stria 8 entirely carinated (Fig. 1D). All described *Epactoides* have nine distinct elytral striae, however, some species show an additional stria on the top of the carina of the 7^th^ stria (see for example *E.frontalis*; Fig. 1J).

Base of elytral interstriae 2–5 tuberculated (Fig. 1E): this trait is quite rare in *Epactoides*, but common in the allied genus *Ochicanthon*. *Epactoideshelenae* (Montreuil, 2005) shows tiny and pointed tubercles on the basal margin of the interstriae 3–6.

Prosternal spur (Fig. 1G): usually, the prosternum of *Epactoides* is simple and rather flat, without central spurs or tubercles, but the prosternum of *E.giganteus* sp. nov. has a well-developed spur in its medial region.

Finally, the holotype of *E.giganteus* sp. nov. has two anteromedial pronotal pits (Fig. 1A, F). Our examination has confirmed that this character is not sexually dimorphic and intraspecifically variable within *Epactoides* (found in *E.frontalis* and *E.lissus* Lebis, 1953) and other Madagascan genera (e.g., *Apotolamprus* and *Arachnodes* Westwood, 1842). We examined if these pits serve as sites of muscle attachment by bleaching the body of some Madagascan *Epactoides*, *Apotolamprus*, and *Arachnodes* in hydrogen peroxide. However, no traces of muscular connections were found. Thus, so far, the anatomical function and systematic informativeness of the pronotal pits remains unknown.

##### Historical notes and hypotheses on the origin of the holotype of *Epactoidesgiganteus*

The holotype of *E.giganteus* sp. nov. was apparently collected by Jean Nicolas Bréon, botanist and then director of the current Jardin de l’État in Saint-Denis (Réunion). Bréon arrived in Réunion in 1817, but health problems forced him to leave the island in 1833 (Bouchard-Huzard 1865). During this period, he undertook numerous expeditions to the southeastern of Madagascar (Anôsy) and Île de Sainte-Marie (northeastern Madagascar). Also, it is known that in Réunion, Bréon occasionally collected insects and sent lots of entomological material to the MNHN. However, mislabeling seems to be common with his specimens. Macquart (1843) was the first author to point out that some of the flies supposedly collected by Bréon in Réunion and sent to the MNHN (accession number 4112.33) were actually mislabeled, as they belonged to common European species [e.g., *Spilogasterquadrivittata*, but see taxonomic corrections by Pont (2012)]. According to Pont (2012), the historical accession catalogue of the MNHN of Paris indicates that the specimens under the accession number 4112.33 belong to a set of insects collected and sent by Bréon from Île Bourbon (ancient name of Réunion island). Beneath this information, in the same catalogue, A.C. Pont found a note from the coleopterist E.J.B. Fleutiaux (1858–1951) saying that “three Elateridae (Coleoptera) labelled as collected on La Réunion by Bréon are in fact European species”. Nonetheless, within the same bunch of insects, there was also the holotype of the melolonthid *Gymnogasterbuphthalma* Blanchard, 1850, which was originally described from a single female. The provenance of this specimen was questioned for more than 130 years, when eventually new specimens were recollected in the north of the island (Lacroix 1988), indicating that *G.buphthalma* is endemic to Réunion.

Given the aforementioned facts, we cannot rule out the possibility that *E.giganteus* sp. nov. has been collected in Madagascar and mislabeled afterwards. On one hand, it is noteworthy to consider that during the last 20 years Madagascan dung beetles have been intensively surveyed (e.g., Hanski et al. 2007; Rahagalala et al. 2009; Viljanen et al. 2010; Knopp et al. 2011) and no other specimens of *E.giganteus* sp. nov. were collected. On the other hand, however, it is important to remark that since 1950, Madagascar lost about 44% of native rainforests (Morelli et al. 2020), which may have caused the disappearance of many forest-dwelling species, including dung beetles (Hanski et al. 2007). Indeed, likewise its congeneric species, *E.giganteus* sp. nov. is suggested to be a putative forest dweller, which may have gone extinct long before the beginning of the intensive dung beetle surveys.

Thus, the limited data we have do not allow to confirm the correct provenance of *E.giganteus* sp. nov. At the same time, we are lacking any direct evidence that could suggest the mislabeling of the holotype of *E.giganteus* sp. nov. Réunion is the island of the Mascarene Archipelago that still preserves the largest amount of forest habitats, with about one-third of its surface covered by native vegetation (Strasberg et al. 2005; Lagabrielle et al. 2011). It is logical to believe that Réunion could (or still can) harbor native dung beetles, likewise other islands of the Mascarene Archipelago (Mauritius: five extant species, Paulian 1939; Vinson 1939, 1946, 1958; Rodrigues: ca 12 undescribed subfossil species, Nicholas Porch unpubl. data). Therefore, based on the aforementioned facts and unique morphological features of the holotype, we consider *E.giganteus* sp. nov. to be the first native dung beetle discovered in Réunion.

Over the last decades, different coleopteran groups from Réunion have been surveyed (e.g., Poussereau et al. 2011; Poussereau 2014; Wanat and Poussereau 2019), but no systematic and geographically extensive monitoring of dung beetles have been carried out so far. To date, only occasional collecting events by hand and without standardized methods (i.e., pitfall baited with excrement) have recorded a few introduced *Onthophagus* dung beetles (Lacroix and Poussereau 2019; Jacques Poussereau pers. comm. to Olivier Montreuil). Hence, there is an urgent need to increase efforts to study scarabaeine dung beetles in Réunion island to confirm or contradict the possibility that today’s still surviving pristine habitats can preserve any endemic species, including *E.giganteus* sp. nov., or even unveil their unknown diversity.

The volcanic Mascarene islands (ca 3–10 Myr) have never been connected to other landmasses, and over-water dispersal is the predominant scenario to explain the origin of animals and plants inhabiting the Mascarene Archipelago (Vences et al. 2003; Yoder and Nowak 2006; Harmon et al. 2008; Buerki et al. 2013; Bukontaite et al. 2015). Importantly, over the last 65 Myr a series of now submerged continental fragments located in the northern part of the Mascarene Plateau fostered the Madagascar-to-Mauritius dispersal route (Bradler et al. 2015; Kehlmaier et al. 2019; Przybyłowicz et al. 2021). As a consequence, Mauritius played as critical source of biota for the geologically younger Réunion (Agnarsson and Kuntner 2012). Cases of direct colonization of Réunion from Madagascar are rare in the literature.

Thus, assuming that *E.giganteus* sp. nov. is native to Réunion and considering the nowadays consolidated hypothesis of Madagascan origin of the genus *Epactoides* (ca. 30–19 Myr) from African ancestors (Wirta et al. 2010), the most likely scenario is that *E.giganteus* sp. nov. colonized Réunion by over-water dispersal from Madagascar.

## Supplementary Material

XML Treatment for Epactoides giganteus
